# Supplementary material for: Maslinic Acid Supplementation during the In Vitro Culture Period Ameliorates Early Embryonic Development of Porcine Embryos by Regulating Oxidative Stress
Source: Animals (Basel). 2023 Mar 13;13(6):1041. doi: 10.3390/ani13061041 (PMC10044061; doi:10.3390/ani13061041)
Supplement: Supplementary file 1 [file animals-13-01041-s001.zip › Figure S1 legend.pdf]

Figure legend

Figure S1. MA supplementation reduces H_2O_2-induced intracellular ROS levels in porcine parthenogenetic embryos. Representative fluorescence images of intracellular ROS in parthenogenetic embryos at the four-cell **(A)** and blastocyst **(B)** stages in the presence or absence of MA under H_2O_2-induced oxidative stress. Scale bar=100 μm. Relative intracellular ROS levels in four-cell **(C)** and blastocyst **(D)** stage embryos. The numbers of embryos examined in each group are shown in the bars. The values are expressed as the mean ± SEM, and significant differences are represented with ** ($p < 0.01$) and **** ($p < 0.0001$).
