# Supplementary material for: Maslinic Acid Supplementation during the In Vitro Culture Period Ameliorates Early Embryonic Development of Porcine Embryos by Regulating Oxidative Stress
Source: Animals (Basel). 2023 Mar 13;13(6):1041. doi: 10.3390/ani13061041 (PMC10044061; doi:10.3390/ani13061041)
Supplement: Supplementary file 1 [file animals-13-01041-s001.zip › Figure S2 legend.pdf]

Figure legend

Figure S2. Relative mRNA expression of *BCL2*, *BAX*, *HO-1* and *DHODH* in porcine parthenogenetic embryos in the control and MA supplementation groups. The data are expressed as the mean ± SEM from at least three separate experiments, and significant differences are represented with *($p < 0.05$), ** ($p < 0.01$), *** ($p < 0.001$), and **** ($p < 0.0001$).
