# Supplementary material for: Maslinic Acid Supplementation during the In Vitro Culture Period Ameliorates Early Embryonic Development of Porcine Embryos by Regulating Oxidative Stress
Source: Animals (Basel). 2023 Mar 13;13(6):1041. doi: 10.3390/ani13061041 (PMC10044061; doi:10.3390/ani13061041)
Supplement: Supplementary file 1 [file animals-13-01041-s001.zip › Table S1.pdf]

Table S1 Sequences of primers used for RT-qPCR

**Gene**	**Forward Primer (5' – 3')**	**Reverse Primer (3' – 5')**
BCL-2	AGGGCATTCAGTGACCTGAC	CGATCCGACTCACCAATACC
BAX	CATGAAGACAGGGGCCCTTT	CATCCTCTGCAGCTCCATGT
DHODH	TGCCTTGCGCTCTGAAACTG	TCTGCTCCAATGGCGTCTGT
HO-1	ACCCAGGACACTAAGGACCA	CGGTTGCATTCACAGGGTTG
GAPDH	AAGTTCCACGGCACAGTCAA	CACGCCCATCACAAACATGG
