# Supplementary figures and images for: Maslinic Acid Supplementation during the In Vitro Culture Period Ameliorates Early Embryonic Development of Porcine Embryos by Regulating Oxidative Stress
Source: Animals (Basel). 2023 Mar 13;13(6):1041. doi: 10.3390/ani13061041 (PMC10044061; doi:10.3390/ani13061041)

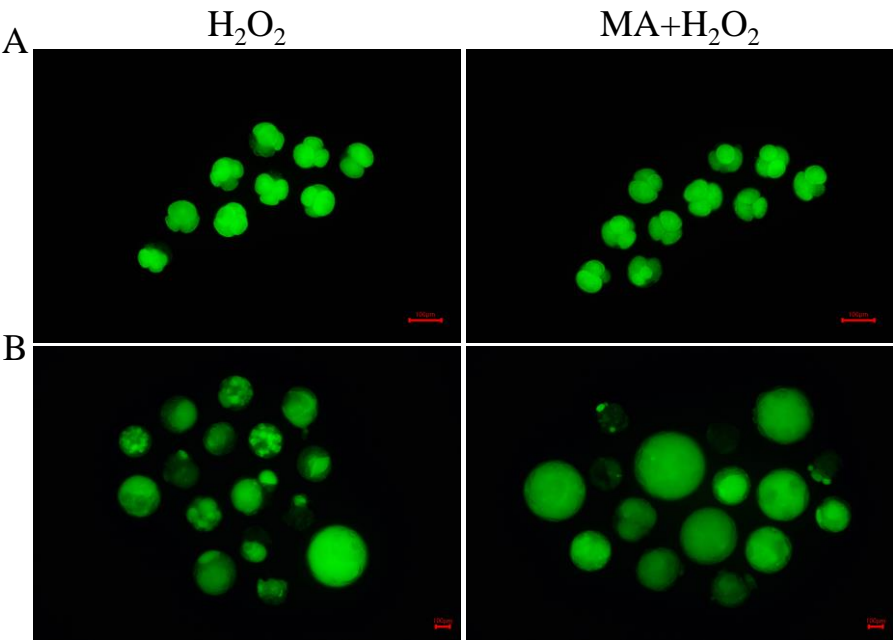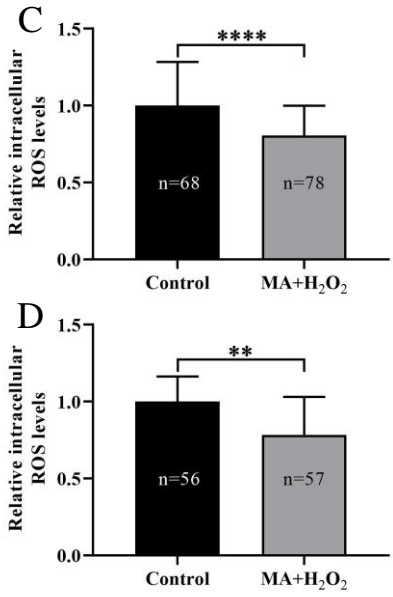

Supplement: Supplementary file 1 [file animals-13-01041-s001.zip › Figure S1.pdf]

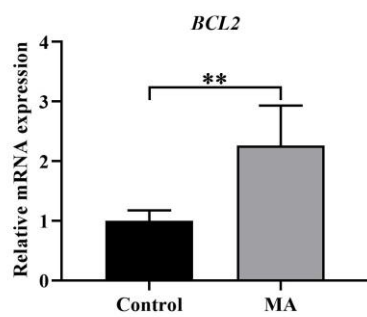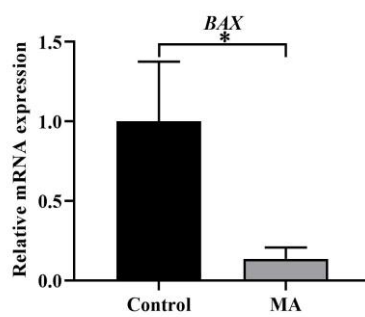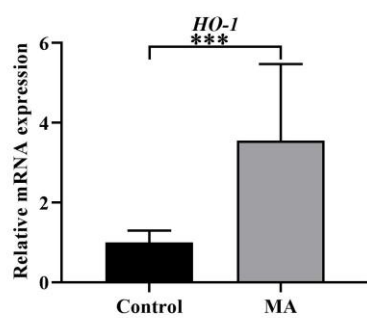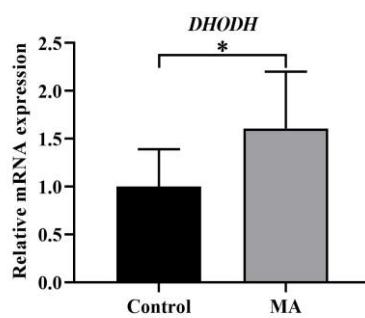

Supplement: Supplementary file 1 [file animals-13-01041-s001.zip › Figure S2.pdf]
